# [Corrigendum] Epigallocatechin‑3‑gallate inhibits nicotine‑induced migration and invasion by the suppression of angiogenesis and epithelial‑mesenchymal transition in non‑small cell lung cancer cells

**DOI:** 10.3892/or.2023.8614

**Published:** 2023-08-09

**Authors:** Jingli Shi, Fei Liu, Wenzhang Zhang, Xin Liu, Bihua Lin, Xudong Tang

Oncol Rep 33: 2972–2980, 2015; DOI: 10.3892/or.2015.3889

Subsequently to the publication of the above paper, the authors have drawn to the attention of the Editorial Office that a pair of the data panels showing the results of wound-healing assay experiments (in Fig. 1A) and Transwell invasion assays (in Fig. 1B) on p. 2975 were inadvertently featured incorrectly in this figure. Specifically, in Fig. 1A, the ‘nicotine + 25 μM EGCG / 0 h’ data panel was erroneously copied across to represent the ‘nicotine + 0 μM EGCG / 0 h’ image, whereas in Fig. 1B, the representative invasion image for the ‘nicotine + 10 μM EGCG’ experiment was also incorrectly placed.

The authors were able to re-examine their original data files, and realize how the errors were made during the assembly of this figure. The revised version of Fig. 1, showing the correct data for the ‘nicotine + 0 μM EGCG / 0 h’ in Fig. 1A and the ‘nicotine + 10 μM EGCG’ experiment in Fig. 1B, is shown on the next page. Note that the errors made in assembling this figure did not affect the overall conclusions reported in the paper. The authors are grateful to the Editor of *Oncology Reports* for allowing them the opportunity to publish this Corrigendum, and all the authors agree with its publication. They also apologize to the readership for any inconvenience caused.

## Figures and Tables

**Figure 1.**
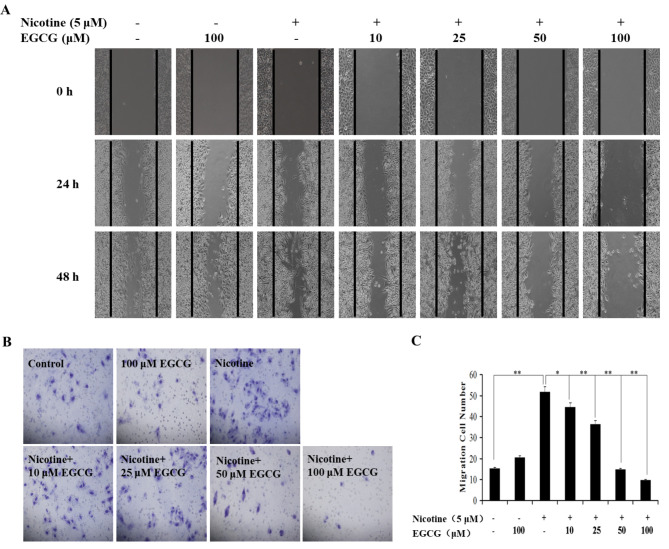
EGCG inhibits nicotine-induced migration and invasion in A549 NSCLC cells. (A) The migration of A549 cells exposed to nicotine in the presence or absence of EGCG was analyzed by a wound-healing assay. (B and C) The invasion of A549 cells exposed to nicotine in the presence or absence of EGCG was detected by the Matrigel invasion chamber. (B) Images are representative of results from three independent experiments. (C) The number of invasive cells from (B). The data are presented as the mean ± SD from three replicate experiments. *P<0.05; **P<0.01. EGCG, epigallocatechin-3-gallate; NSCLC, non-small cell lung cancer.

